# Fostering diversity and a culture of inclusion in clinical research by training and hiring community members as clinical research assistants

**DOI:** 10.1017/cts.2022.480

**Published:** 2022-10-28

**Authors:** Katelyn Collinger, Laurel L. Yasko, Denise Smalley, Kimberly Rassau, William E. Strickland, Steven E. Reis

**Affiliations:** 1 Clinical and Translational Science Institute, University of Pittsburgh, Pittsburgh, PA, USA; 2 Manchester Bidwell Corporation, Pittsburgh, PA, USA

**Keywords:** Clinical research assistant, workforce diversification, research staff training, workforce development, community engagement

## Abstract

The University of Pittsburgh (Pitt) Clinical and Translational Science Institute (CTSI) and the nonprofit Bidwell Training Center co-developed a new program for translational workforce diversification and development to foster diversity and inclusion in clinical research. The STricklAnd Research Training (START) program provides students in the Medical Assistant program at Bidwell a career path in clinical research. We created a 12-hour didactic package that covers responsible conduct of human subjects research and good clinical practice as an add-on to existing vocational curriculums. Students have the option of completing a clinical research-related externship at Pitt, which includes mentoring, shadowing, and protocol-specific training on a study team whose intention is to hire them as a clinical research assistant. Those who accept a position at Pitt receive continued mentorship, education, and professional development through Pitt CTSI. In the first three cohorts, two of which had access to research externships at Pitt, 92% of students successfully completed the instruction in clinical research. We plan to expand START to new venues to train and hire local community members from diverse backgrounds who can bring their lived experience to research programs.

## Introduction

Over its four funding periods, the University of Pittsburgh (Pitt) Clinical and Translational Science Institute (CTSI) has developed numerous educational initiatives for students, trainees, staff, and investigators at Pitt. However, we recognized an unmet need to address the ongoing shortage and turnover of research staff as well as the lack of diversity.

In parallel, CTSI has also been committed to embedding research reciprocity as a guiding core principle in translational research [1]. We embarked on initiatives to actively integrate community members in all aspects of the research process, such as Community-Partnered Research Ethics Training [2] to allow community members to participate as coinvestigators in individual studies, but we had not specifically considered training and hiring community members into permanent research staff roles.

As part of CTSI 4.0, we developed a pathway through which members of local underrepresented communities can be trained and hired to work at Pitt as clinical research assistants, thus providing a durable bridge from community to academic research. This initiative arose out of a discussion at a CTSI External Advisory Board meeting during which Board member William E. Strickland, founder and then President of the nonprofit Manchester Bidwell Corporation, pointed out that graduates from his Bidwell Training Center programs could help fill research staff vacancies and help diversify clinical research teams.

For over 50 years, the Bidwell Training Center, an accredited, state-licensed, nonprofit job training center with established roots in a marginalized community, has provided career training at no cost to adults in transition. Bidwell receives funding from the Commonwealth of Pennsylvania, private foundations, and individual donors, making it possible for all students to receive full tuition, textbooks, uniforms, and supplies required for training at no cost to them. Bidwell offers career-oriented, tuition-free training in six majors, three of which are in allied health fields. All Bidwell trainees also complete a practical externship with potential employers.

We created the ***STricklAnd Research Training*** program, named in honor of Mr. Strickland, to provide Bidwell students a career path in clinical research through didactic training, externships, mentoring, job placement, and continuing education and professional development. This program is also part of our initiative to actively infuse inclusion, diversity, equity, and access (IDEA) into all aspects of the research process at Pitt, including research teams themselves. A major goal of our IDEA initiative centers on workforce diversification at all levels, and the START program will contribute to successful achievement of this goal.

## STricklAnd Research Training (START) Program

### Competency-Based Curriculum

Three of us (KC, LY, DS) worked with Bidwell Training Center to develop competency-based clinical research training to be incorporated within Bidwell’s Medical Assistant program. We chose the Medical Assistant program as the foundation for our START curriculum because students learn other skills during the 7-month program that are also essential for clinical research assistant positions, such as phlebotomy, electronic health records, vital signs, and patient interactions.

We drafted the START curriculum based on the main topics in four Collaborative Institutional Training Initiative (CITI) modules: Responsible Conduct of Research, Privacy and Information Security, Good Clinical Practice, and Human Subjects Research. Initially, the training comprised 2 weeks of pre-recorded presentations that the students watched, followed by the completion of homework assignments, and attending a remote meeting with the section presenters. Student were then asked to complete three CITI modules: Responsible Conduct of Research, Privacy and Information Security, and Human Subjects Research.

We received feedback from the students and the Medical Assistant Instructor at Bidwell Training Center that this was too time-consuming and dense. We therefore revised the didactic training for second cohort to cover eight sessions (Table 1) in six weekly classes, each lasting 2 hours. The students attended 1-hour remote lectures during which section presentations are delivered live, followed by questions and discussion. The program is currently delivered remotely using Google Classroom.

**Table 1.** STricklAnd Research Training (START) program competencies

Section	Competencies
Overview of the clinical research process and common research terminology	Describe the different types of clinical research study designs Describe the phases involved in the drug development process Identify the main components of a research protocol Describe the difference between medical procedures and research procedures
Good clinical practice and other regulations	Demonstrate basic knowledge of the Belmont Report, Code of Federal Regulations, and the International Council for Harmonization’s Good Clinical Practice Describe the role of the Institutional Review Board (IRB)
Privacy, confidentiality, and information security	Explain the difference between privacy and confidentiality and methods to protect each in the research setting
Overview of study documentation	Demonstrate knowledge of required clinical trial documents
Research misconduct	Demonstrate knowledge of the different types of research misconduct, factors that can lead to misconduct, and how to prevent, mitigate, and detect research misconduct
Recruitment and retention in research	Demonstrate an understanding of how recruitment and retention are defined in research Demonstrate an understanding of the challenges surrounding recruitment and retention and how to identify tools to help overcome barriers Demonstrate an understanding of federal and local regulations regarding allowable practices for recruitment and retention
Informed consent process	Demonstrate an understanding of informed consent, the process of obtaining informed consent, and ongoing informed consent discussions Understand the importance of cultural sensitivity, diversity, and inclusivity in research Using a research study example, demonstrate ability to describe study to potential participants (study pitch)
Intro to research lab regulations and procedures	Demonstrate a basic understanding of the biosafety levels in a laboratory Demonstrate a basic knowledge of laboratory equipment and maintenance Demonstrate basic knowledge of general lab safety
CIRTification online: community involvement in research training	Introduction to Research: basic terminology, activities, and people involved in research and various ethical considerations Research History: past research abuses, rules to prevent these abuses, goals of community engagement in research, and the important responsibilities shared by community research partners Eligibility and Recruitment: importance of adhering to study inclusion/exclusion criteria and best practices for identifying and recruiting research participants Informed Consent: key components of informed consent, types of information included in the consent form, how to respond to participant questions about enrollment and withdrawal, and discussion of practical challenges and good practices Institutional Review Board: people on the IRB, process of submitting protocols to the IRB, and criteria that IRB applies to review of the research Collecting and Protecting Data: how to protect participant privacy and keep data safe and confidential during collection, transport, and storage Handling Issues in the Field: what do to if co-worker suspected of falsifying data, meet a study participant outside of work, and are asked challenging questions from study participants

Instead of homework, short quizzes are given to check comprehension. Successful completion of the training is based on attendance, participation, quiz scores, a role-playing exercise, and – instead of CITI modules – completion of CIRTification Online: Community Involvement in Research Training [3], which is a free, interactive web-based human research protections training program tailored specifically for community research partners. This approach was better received by our second and third cohorts (Table 2) and will be continued going forward.

**Table 2.** START program summary data

Cohort	November 2020	May 2021	November 2021
Medical assistant students*	8 (7 female; 4 White, 1 Black, 2 Hispanic, 1 unknown; 20–43 years)	14 (13 female; 5 White, 7 Black, 1 Black/Asian, 1 White/Black, 1 Hispanic; <21–51+ years)	14 (10 female; 7 White, 6 Black, 1 White/Black; <21–51+ years)
Passed START**	7	14	12
Research externs	***	4 (4 female; 2 White, 2 Black; 2 21–30 years, 2 51+ years)	4 (2 female; 2 White, 2 Black; 1 < 21 years, 2 21–30 years, 1 51+ years)
Pitt hires	***	2 (2 female; 2 Black; 2 51+ years)	2 (1 female; 2 White, 1 < 21 years, 1 51+ years)

* Total number enrolled in Bidwell Training Center Medical Assistant program.

** Satisfactory completion of quizzes, role-playing, participation, CIRTification.

*** Externship agreement not yet executed at time of first cohort, no job opportunities as result.

### Externship with mentoring and shadowing

A key component of all Bidwell programs is an externship, which must be unpaid per accreditation requirements. Students in the START program have the option of completing a clinical research-focused externship through the CTSI, during which students receive protocol-related training and mentorship. The importance of this type of hands-on externship for research staff is highlighted by recent findings that clinical research coordinators found experience, day-to-day practice, and observing colleagues and mentors an essential supplement to formal Good Clinical Practice training [4].

To enable START trainees to perform their externship at Pitt, an agreement was signed between the University of Pittsburgh on behalf of the CTSI and Bidwell Training Center. Externship experiences are study-specific and include shadowing in successful research programs and rotating through the CTSI Clinical and Translational Research Program sites, which provide specialized data collection and intervention services.

We also created a Memorandum of Understanding signed by CTSI and research groups interested in hiring a START program graduate as a research assistant. This Memorandum outlines expectations, including providing the START trainee with a hands-on externship opportunity, mentoring both through CTSI and the research program, and employment following successful completion of the externship. Although the externship is required to be unpaid, we were able to give START trainees vouchers for parking and bus fare.

We work with selected clinical research teams to create externships through which Bidwell students can learn and practice essential skills, such as recruiting patients for research studies, obtaining informed consent, and administering assessments. Because we specifically place students with research groups seeking to hire a clinical research assistant, the externship experience can also serve as initial job training and help ensure that the personnel fit is good. We plan to offer research teams participating in the externship bias reduction and other training in advance to ensure an inclusive, welcoming, and supportive culture and environment.

START externs must successfully complete CITI modules required for clinical research. Extern competencies are evaluated at weeks two and four of the externship, and, to date, all externs have passed their mid-review and final competency evaluations. Should a future extern have difficulty with one or more competencies tested, we would work with that START graduate and with Bidwell Training Center to adjust the externship to address the needed competency.

### Transition to employment

As noted above, students are matched with a clinical investigator who has a research assistant job opening, with the intention that the externship will transition to full employment at Pitt. Working with the clinical study team as part of their formal training ensures START trainees are fully aware of what is expected of them when they are hired. At the same time, clinical investigators are briefed in advance about their expectations for START program students, since they do not come from an undergraduate background like most clinical research assistants.

A Workforce Development Career Counselor in Human Resources works with the START program students to help them prepare, submit, and track their job applications. The counselor can also help ensure the applications are reaching the research teams.

### Career advancement

Research staff at all career stages have professional development opportunities through CTSI, and all employees at Pitt receive tuition benefits that would allow START graduate hires and their spouses and children to pursue college degrees. The Pitt College of General Studies offers a variety of learning options designed for adult and non-traditional students and includes flexible scheduling for those who work full-time.

Research teams who hire START graduates are specifically asked to give these research assistants time off to attend the CTSI Introduction to Research Workforce activities, which includes the 2-day Orientation to Research Fundamentals workshop offered semi-annually, as well as CTSI Diversity, Equity, and Inclusion trainings. In addition, we offer more advanced workshops and trainings throughout the year that START hires can complete to document new skills that could qualify them for salary increases. Following hire, the START program coordinator routinely checks in with the START graduate to provide mentoring and support as they transition into their new role as a clinical research assistant.

### Program enrollment

Students enroll in the Bidwell Medical Assistant program and then learn about the START training. Since November 2020, three cohorts have completed the START program (Table 2). The first cohort had lower enrollment numbers than is typical for the Bidwell Training Center due to the COVID-19 pandemic; typical class size is 14–20. Due to a delay in executing the externship agreement, Pitt was not able to host the first cohort of externs after they completed their START training.

All 14 students in the second cohort successfully completed both the medical assistant and START training. All four students who chose to complete their externships through CTSI had the opportunity to apply to open research assistant positions at Pitt. Three out of the four applied and were offered positions, and one student accepted the job offer at Pitt; one of the other students initially decided not to accept the offer but later came back to apply again and has been hired. In the third cohort, four START trainees completed their externship through CTSI. Two pursued clinical research assistant jobs at Pitt, while the other two students decided to pursue other careers.

To date, START graduates hired at Pitt have been majority female and older (>51 years), and half (2/4) are Black. Table 3 provides quotes from START externs and hires on why they did or did not pursue a career in research and, for current hires, their experience.

**Table 3.** Qualitative feedback on decision regarding research career

Extern did not pursue research position	Extern hired as research assistant at Pitt
Decided to get a job (outside of research and medical assisting) at more convenient location due to child care needs – plans to move out of state	My experience at All Of Us has been ongoing training, which is great. I am trained in research regulation, responsible conduct in research, confidentially, enrollment and retention, and the IT platform. I am visiting other All Of Us sites to complete participants' enrollment in the program and performing phlebotomy and biometrics. I know how to follow proper protocol in research.
Applied for research position but was not hired due to transportation concerns – got a position as a medical assistant with Allegheny Health Network	
Extern indicated not knowing what he wanted to do following graduation	So far my experience is good here at Pitt. My team is helpful during my training. They had two check-in meetings with me in the last month. It is just a lot for me to learn.
Extern decide to go with another job offer outside of research – no reason given	

### Future plans

We are evaluating the success of the program itself quantitatively and qualitatively. At the conclusion of the didactic training and again at the conclusion of the externship, Bidwell students complete an evaluation form, and their feedback is used to improve the START program.

For those hired at Pitt, we will monitor whether START graduates remain in research assistant positions at Pitt longer than more traditional hires with college backgrounds, since the START research assistants may be more likely to view their positions as career opportunities with room for advancement. We will also monitor whether they pursue a college degree on a career path to become a biomedical researcher.

We will similarly evaluate the quantitative (e.g., enrollment-retention of study participants from underrepresented backgrounds) and qualitative (e.g., team perspectives on inclusion and awareness of bias) impact of START graduates on the clinical studies and the research teams in which they are involved.

To enhance retention, we will create a near-peer mentoring program partnering START program hires with experienced research staff. The near-peer mentor will be trained by Master Facilitators at Pitt who are certified in teaching the Entering Mentoring curriculum [5]. The near-peer mentor will meet with their START mentee monthly or more frequently if needed.

To support career advancement, we are working closely with Human Resources and the Office of Diversity at Pitt to create a competency-based structure for research staff that can be used for hiring, promotion, and compensation. In collaboration with the Duke CTSA Hub, we are adapting for use at Pitt their REDCap tool to identify job classifications based on competencies. Duke’s tool enables faculty to identify potential staff and list the job responsibilities for that role, and then map the role to a job title [6,7]. The program creates a professional pathway, identifies advancement opportunities, and defines objective assessments of competencies needed for advancement. In parallel, we will adapt our online Customized Career Development Platform to enable research staff to strategically plan their careers by identifying career goals and linking competency-based training that is available at Pitt or nationally to each goal. This competency-based pathway for career advancement will help START Program hires clearly understand what they need to learn or accomplish to be eligible for a step increase in salary within their job classification, and CTSI will be available to help them track progress and achieve these goals.

## Discussion

The START program was created as a partnership between CTSI and the nonprofit Bidwell Training Center to expand the career opportunities of medical assistant trainees from disadvantaged backgrounds as clinical research assistants. The START program also serves to diversify Pitt’s translational research workforce in terms of background and lived experience, and our ultimate goal is for translational research at Pitt to be enriched at all stages – from the formation of research questions through dissemination of results back to the community – through the diversity of life perspectives brought by START graduates to study teams.

We have and will continue to learn from these students as we refine the START program. We launched START just prior to the COVID 19 pandemic, which both reduced the class size for each START cohort and guided our decision to deliver the clinical research training remotely. However, the remote training made continued engagement and rapport building with the students difficult. Once it was safe to do so, we began visiting the Bidwell Training Center in person after students completed the didactic training but before they indicated their choice for externship placement. We had a clinical research assistant from Pitt host a question and answer session at which they spoke about their background, what led them to research, and what their job entails and to answer Bidwell student questions. For the most recent cohort, we were able to have a START program graduate currently employed at Pitt return to Bidwell to talk with their peers.

In addition, we learned that it is imperative to talk with the students about job requirements and location. This could include whether the position is part-time or full-time, whether job-related travel is required, and whether the job location(s) are located on a bus line or have parking available. We also recognized the importance of confirming that each student’s experience and education fulfill job requirements to ensure that the student is a good fit for the position and vice versa.

A major challenge that arose is the pandemic-related staffing shortage in all areas of clinical care. Prior to COVID-19, research assistant salaries were similar if not slightly above typical medical assistant salaries. Starting in 2020, however, private employers began offering sign-on bonuses and higher wages, making academic positions less competitive. Our current approach in recruiting is to emphasize the benefits that academic institutions have to offer beyond salary, including good insurance and retirement plans, tuition benefits for employees and their families, and generous leave policy. We also emphasize the career advancement and continuing education opportunities that academic positions offer. We plan to keep in touch with START graduates to ensure they realize the door is always open at Pitt should they later become interested in a research assistant position. Indeed, one student who initially turned down a position at Pitt later returned and was hired.

Our implementation of the START program in three cohorts has allowed us to refine the didactic component, identify and address challenges, and set the stage for expansion. Our most important outcome is reflected in the percentage of students who successfully completed the START program as part of their Bidwell training (92%) and who will therefore carry with them a strong understanding of and comfort with clinical research, no matter where they work. Although not every trainee will choose to pursue a career in research, they can serve as ambassadors on behalf of clinical research and reinforce the importance and value of research participation to others in their communities. For those who accept research assistant positions at Pitt, we look forward to evaluating their impact on the research teams with whom they work and the research studies they help support.

Looking toward scaling the program and program replication, we will develop and disseminate an educational package with our eight training sessions, comprehension quizzes, and online CIRTification as an add-on to any standard medical assistant program. The parent nonprofit organization of Bidwell Training Center, the Manchester Bidwell Corporation, supports a network of similar training centers throughout the US, including 13 located near CTSA Hubs, which offers the opportunity for national dissemination of our program throughout the CTSA Consortium.

We are also exploring additional potential partners to determine if the START program can be adapted for use in other vocational program and community college settings, similar to the Clinical Research Coordination Certificate offered by Urban College of Boston [8]. In addition, we will consider developing community-based training programs for other critically needed research staff positions, including laboratory technicians, data entry and coding, and call centers.

In summary, the CTSI START Program addresses multiple needs simultaneously. First, there is the economic opportunity and new career option afforded to local residents seeking to advance their earning potential but for whom 2- or 4-year college is not an option, at least not initially. Second, filling research staff positions with locally trained community members advances biomedical research workforce diversification in terms of both demographics and personal and socioeconomic perspectives and backgrounds. Third, members of underrepresented communities hired into the Pitt research ecosystem will bring important new voices and experiences to diversity, equity, and inclusion initiatives at CTSI and across the university. Fourth, creating a career pathway and providing career planning and development support for locally trained community members may help reduce staff turn-over, which can significantly affect the ability of an investigator to efficiently complete a research study on time. Finally, hiring community members as research assistants further promotes our goals of both research reciprocity and research receptivity, which we believe will translate to improved recruitment and retention of participants from underrepresented backgrounds.
